# Application of Polysaccharide-Based Hydrogels as Probiotic Delivery Systems

**DOI:** 10.3390/gels4020047

**Published:** 2018-05-22

**Authors:** Iwona Kwiecień, Michał Kwiecień

**Affiliations:** 1 Department of Physical Chemistry and Technology of Polymers, Silesian University of Technology, M. Strzody 9, 44-100 Gliwice, Poland; 2 Centre of Polymer and Carbon Materials, Polish Academy of Sciences, M. Curie-Skłodowskiej 34, 41-819 Zabrze, Poland; mkwiecien@cmpw-pan.edu.pl

**Keywords:** polysaccharides, hydrogels, probiotics, alginate, κ-carrageenan, xanthan, pectin, chitosan

## Abstract

Polysaccharide hydrogels have been increasingly utilized in various fields. In this review, we focus on polysaccharide-based hydrogels used as probiotic delivery systems. Probiotics are microorganisms with a positive influence on our health that live in the intestines. Unfortunately, probiotic bacteria are sensitive to certain conditions, such as the acidity of the gastric juice. Polysaccharide hydrogels can provide a physical barrier between encapsulated probiotic cells and the harmful environment enhancing the cells survival rate. Additionally, hydrogels improve survivability of probiotic bacteria not only under gastrointestinal track conditions but also during storage at various temperatures or heat treatment. The hydrogels described in this review are based on selected polysaccharides: alginate, κ-carrageenan, xanthan, pectin and chitosan. Some hydrogels are obtained from the mixture of two polysaccharides or polysaccharide and non-polysaccharide compounds. The article discusses the efficiency of probiotic delivery systems made of single polysaccharide, as well as of systems comprising more than one component.

## 1. Introduction

Nowadays, people’s awareness of importance of health is significantly increasing. Consumers are more often interested in food products having positive impact on their health. This type of functional food is enriched with components, such as vitamins, antioxidants, proteins or probiotics, providing benefits other than just nutrition [1,2]. Unfortunately, many bioactive components used in the food industry are sensitive to the manufacturing process and storage conditions. Exposing them to, e.g., oxygen, elevated temperatures, certain pH or light, might be harmful [3]. Therefore, delivery systems for bioactive components are designed to protect probiotics against adverse conditions. At some point, biomaterials used in food products as the delivery systems will come into contact with human digestive tract. They have to meet several requirements, such as non-toxicity, compatibility with bioactive components and relatively low cost [4]. Numerous polysaccharides can meet those requirements. In this review, we focus on polysaccharides-based hydrogels used as delivery systems for probiotics. Probiotics are sensitive to elevated temperatures, consequently entrapment of probiotic cells in hydrogel matrix has to proceed under mild conditions. The ionic gelation method, which does not require harsh conditions, has been suitable for obtaining hydrogel probiotic delivery systems. Moreover, the ionic gelation as physical cross-linking method, allows avoiding potentially toxic crosslinking agents [5].

According to Food and Agriculture Organization of the United Nations and World Health Organization, probiotics are “live microorganisms which, when administered in adequate amounts, confer a health benefit on the host” [6]. In general, probiotics are associated with having positive effects on the human gastrointestinal tract. However, probiotics also offer other health benefits, for example, they enhance immunity [7], decrease cholesterol levels [8] (and, as a result, control hypertension [9]), or even prevent atopic eczema in infants [10]. Moreover, the use of probiotics might prevent colon cancer [11] or reduce breast cancer risk [12]. Because the level of probiotic microbial strains in human intestines might decrease for various reasons, such as unhealthy eating habits, stress or antibiotic therapy [13,14], probiotics should be administered regularly, e.g., with food or pharmaceutical formulations. Unfortunately, the viability of probiotics might be negatively affected during food processing and storage [15]. Moreover, to fulfill their role, probiotics must survive in the acidic conditions in stomach and be delivered to the intestines at high numbers [16]. Therefore, there is a need to develop delivery systems for the probiotic microbial strains which will improve their viability under the gastrointestinal tract conditions, as well as during the storage of the probiotic food products [17]. For probiotic delivery systems, biomaterials such as proteins (gelatin, casein or whey proteins), as well as synthetic polymers, such as poly(d,l-lactic-*co*-glycolic acid), polyvinyl alcohol or Eudragit (poly(methacrylic acid-*co*-ethyl acrylate) 1:1) could be used [18,19,20]. However, the most commonly used materials for encapsulation of probiotic cells are polysaccharides.

**Figure 1:**
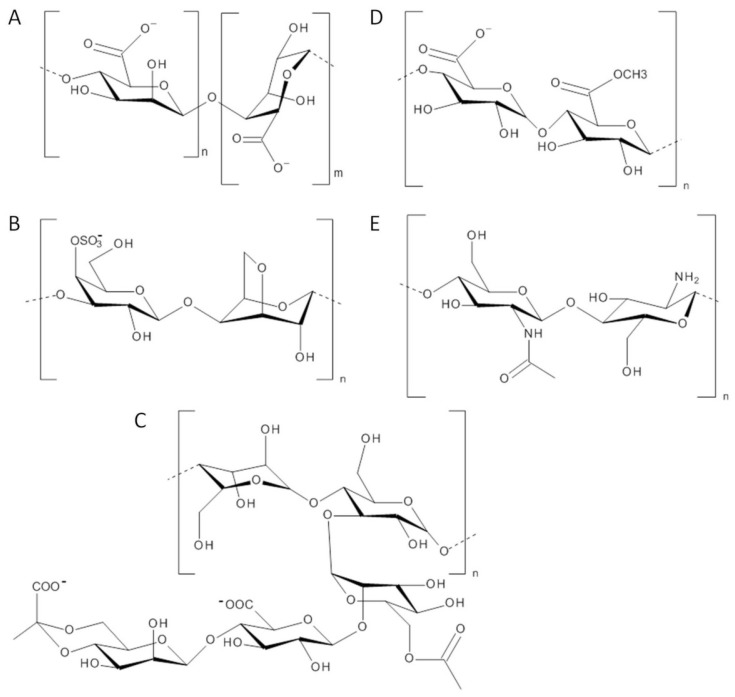
The chemical structures of: alginate (**A**); carrageenan (**B**); xanthan (**C**); pectin (**D**); and chitosan (**E**).

As it is known, the bioactive compound should be retained within delivery system and the system should be stable, until it is exposed to a certain set of environmental conditions. Those environmental conditions (e.g., temperature, pH and enzyme activity) should trigger the release of bioactive compound. Therefore, biomaterials used in delivery systems for probiotics have to be stable in acidic conditions in the stomach and decomposition of such biomaterials should occur only after subjecting them to the small intestine’s pH or pancreatic enzymes [21]. Numerous polysaccharides meet those requirements, for example alginate, carrageenan, xanthan, pectin or chitosan (Figure 1). Hydrogels made of polysaccharides might be used as delivery systems for probiotics, when pore sizes of the hydrogels are adequately small compared to the dimensions of bacteria cells. It ensures entrapment of probiotic cells in hydrogel matrix until the breakdown of the network [22].

## 2. Polysaccharide Hydrogels

### 2.1. Alginate-Based Hydrogels

Alginate is an anionic linear polysaccharide, composed of β-(1,4) linked β-d-mannuronic acid and α-l-guluronic acid residues. Alginate is produced on an industrial level via extraction from the cell walls of brown algae [23]. Composition and sequence of the chains of this polysaccharide might vary depending on algal species or part of the algae used as a source [24]. In addition, biosynthesis, which allows obtaining alginate with more defined chemical structures than alginate extracted from algae, has been considered as promising route for production of this polysaccharide [25]. Alginate, as a non-toxic, biocompatible and biodegradable polymer, has found multiple medical-related applications, such as drug delivery systems, wound dressing or tissue engineering [26]. In many of those applications, alginate has been used in the form of hydrogels [27]. The gelling properties of alginate are related to interaction of α-l-guluronate residues with multivalent cations (e.g., Ca^2+^) [28]. Alginate hydrogels are insoluble in acidic media [29], therefore they can be utilized as delivery systems able to provide protection for the probiotics against acidic gastric juice. The behavior of ionically crosslinked alginate hydrogels, as well as other ionically crosslinked polysaccharide hydrogels, is strongly depends on the pH of the surrounding solution [30]. Calcium alginate hydrogel beads remain stable in the simulated gastric fluid (pH 1.0) and acquire a percent swelling of 110%, while under simulated intestinal fluid (pH 7.4) exhibit a percent swelling over 600%, start to disintegrate and subsequently they dissolve completely [31].

Alginate has been used to prepare multilayer hydrogel beads protecting probiotic bacteria *Bifidobacterium breve* against simulated gastric juice low pH conditions. Probiotic cells were encapsulated in spherical, smooth-surfaced calcium alginate beads using emulsion method. The obtained beads with encapsulated probiotic cells, as well as free cultured cells, were subjected to various pH environments, which simulate gastric juice and intestinal fluids. The viability of *B. breve* cells encapsulated in calcium alginate beads compared with viability of free *B. breve* cells was significantly enhanced [32]. Single layer alginate beads might not provide proper protection for the encapsulated cells against acidic conditions, due to pore size of this hydrogel. However, with the increasing number of layers in alginate beads, the survival of encapsulated probiotic cells has been enhanced [33]. Moreover, such multilayer calcium alginate hydrogels degraded slowly under conditions simulating small intestinal fluid (pH 6.8) and rapidly under conditions simulating colonic fluid (pH 7.2) [34]. Calcium alginate hydrogels improve viability of tested probiotic cells under acid conditions while undergoing degradation under intestinal conditions allowing for the release of encapsulated probiotic cells.

The survivability of bacteria after encapsulation depends on type of strain used in the research, which is associated with different natural resistance of the microorganisms against acidic conditions [35]. Taking that into consideration, calcium alginate hydrogels were applied in encapsulation of other probiotic strains, e.g., *Lactobacillus rhamnosus* and *Lactobacillus acidophilus*. Both strains of bacteria, encapsulated in alginate micro beads and alginate macro beads, as well as free cells, have been subjected to simulated gastric and intestinal fluids. Alginate micro beads were produced by double aerosol method, while alginate macro beads were produced by extrusion method. The *L. acidophilus* was found to be more resistant to acid and bile salts (steroid acids secreted into the lumen of the intestine) than *L. rhamnosus*. Alginate micro and macro beads provide similar protection for *L. rhamnosus* subjected to simulated gastric and intestinal fluids, whereas *L. acidophilus* encapsulated in alginate macro beads showed higher tolerance against acid and bile salts than *L. acidophilus* encapsulated in micro beads. However, protection abilities of micro beads have been further enhanced by coating the beads with chitosan (aminopolysaccharide derived from chitins [36]). As a result of such treatment, diffusion of the acid medium into porous hydrogel matrix has been restricted, and thus the contact between probiotic microorganisms and harmful medium has been limited [37]. The influence of coating alginate microgels with chitosan on the viability of various probiotic strains has been investigated in a study in which alginate microcapsules were prepared using single-stage and double-stage method. In the first method, microcapsules have been obtained in a solution containing crosslinking agent (CaCl_2_) and chitosan, therefore Ca^2+^ and protonated amine groups of chitosan competed to interact with negatively charged carboxylic groups of alginate. In the double-stage method, gelation process was carried out first and the microcapsules have been coated with chitosan subsequently. Encapsulated *Lactobacillus plantarum* using the double-stage procedure in calcium alginate coated with chitosan showed improved viability under simulated gastrointestinal conditions in comparison to free cells and to cells encapsulated by single-stage procedure. In the case of microcapsules obtained by single-stage method, it was found that too close proximity of the chitosan layer to the protected cells has negative effect on cell viability. That effect was explained by increased antimicrobial activity of chitosan resulting from presence of protonated amine groups in chitosan under acidic conditions [38].

Probiotic bacteria are mainly administered with dairy products, however, people with lactose intolerance have to find alternative sources. Fruit juices could serve as an alternative but, having usually a highly acidic pH, they are considered a harmful environment for probiotic cells [39]. Because alginate hydrogels have been proved as suitable materials to enhance the survival rate of probiotic under acidic gastric conditions, their use in protecting bacteria against low pH occurring in fruit juices has been investigated. Selected probiotic strain *L. plantarum* was encapsulated into uncoated alginate beads and alginate beads single and double coated with chitosan. Prepared coated and uncoated beads, as well as free probiotic cells, were kept in pomegranate juice for six weeks at 4 °C (refrigerated storage). Free cells and cells encapsulated into uncoated beads died after four weeks of storage, while cells in single and double coated beads survived six weeks of storage [40]. It might be concluded that chitosan layer contributes to the formation of a thicker, less porous membrane, which impedes penetration of acidic medium into beads.

Delivery systems for probiotics must improve the survival rates during gastric transit as well as to improve the heat tolerance. Thermal treatments, such as the pasteurization process, are often applied during manufacture of food and beverage products [41]. Various strains of probiotic bacteria, namely *L. rhamnosus*, *B. longum*, *L. salivarius*, *L. plantarum*, *L. acidophilus*, *L. paracasei* and *B. lactis*, have been encapsulated in calcium alginate beads using emulsion method and exposed to heat (65 °C). Heat tolerance of encapsulated cells was compared with heat tolerance of free cells. After 30 min of incubation at 65 °C, encapsulated bacteria showed higher rate of survival than free cells, whereas, after 60 min of incubation, the survival rates of free and encapsulated probiotic bacteria were almost similarly low. Those results indicated that calcium alginate beads improve heat tolerance of encapsulated bacteria over limited period. In addition, encapsulated probiotic cells subjected to acidic conditions and bile salts maintained higher viability than free cells subjected to the same conditions [42].

Alginate hydrogels uncoated or coated with chitosan, could be used in probiotic delivery system, as it was demonstrated in research results described above. Moreover, it seems possible to prepare hydrogels efficient for such applications by combining alginate with other non-polysaccharide biopolymers. Whey proteins (mixture of globular proteins, mainly β-lactoglobulin and α-lactalbumin [43]) could be applied as coating material for calcium alginate beads. *Lactobacillus plantarum* strain encapsulated in such beads has been subjected to simulated gastric and intestinal fluid. Results indicated that bacterial survival rate in alginate beads coated with whey proteins has been improved in comparison to uncoated alginate beads [44].

Proteins isolated from pea were combined with alginate to prepare hydrogel beads via extrusion. Shelf life of *Lactobacillus casei* encapsulated in such beads has been tested during storage under different temperatures: 22, 4, and −15 °C. Chosen temperatures correspond to storage conditions at room temperature, in a refrigerator and in a freezer, respectively. Viability of encapsulated cells was compared with free *L. casei* cells. Survival rate of encapsulated bacteria stored at −15 °C was the highest among all samples. Free cells at that temperature might be damaged by forming ice crystals, while hydrogel capsules provided a physical barrier between cells and the ice crystals. Viability of encapsulated cells stored at 22 °C and 4 °C has been disturbed by moisture entering the sample tubes during samples withdrawal [45].

In other studies, negatively charged alginate has been combined with another positively charged biomaterial–elatin (the denatured collagen [46]). The probiotic strain *Lactobacillus salivarious* has been encapsulated in alginate and alginate–gelatin microgels using electrostatic microencapsulation unit and subjected to simulated gastrointestinal conditions. Viability of probiotic cells encapsulated in both types of microgels was compared with viability of free *L. salivarious*. Encapsulated and free probiotics subjected to artificial saliva maintained their viability, while the survival rate of encapsulated probiotics after incubation under simulated gastric conditions has been greatly improved in comparison to the free cells. What is noteworthy, alginate microgels subjected to simulated intestinal fluids have partly dissolved and alginate–gelatin microgels have slightly swelled under those conditions. Such behavior is desired, because it enables the release of probiotic from microgels, and probiotics need to be released to fulfill their role in the large intestine. In addition, to verify survival rates during long-time storage in aqueous-based commercial products, free and encapsulated *L. salivarious* were stored for five weeks in the wet-state. After storage period, viability of encapsulated cells was significantly higher than viability of free cells. Moreover, the thermal stability of encapsulated cells has been tested. The encapsulation allowed for maintaining a higher number of viable cells after heat treatment, simulating thermal processing used in the food industry. Alginate–gelatin microgels were found to be more effective at protecting probiotics during storage, heat treatment, and under simulated gastrointestinal conditions, than microgels based only on alginate. That has been attributed to differences in the physicochemical and structural properties of interior of both types of hydrogel [47].

As can be seen from the above examples, the alginate-based hydrogels have been proven as suitable materials for orally administered delivery systems of probiotic bacteria. All discussed hydrogels are summarized in Table 1. Table 1 also contains information about tested probiotic strains and type of tested conditions. Alginate hydrogels have to be coated, e.g., with chitosan or proteins, to provide proper protection for encapsulated probiotic cells subjected to harmful conditions. However, as was proven in the cited studies [32,33], the alginate beads consisting of several layers are able to efficiently protect encapsulated bacteria against acidic conditions.

### 2.2. Carrageenan-Based Hydrogels

Carrageenan are linear anionic polysaccharides obtained from certain algae species, consisting of alternate units of β-d-galactose and 3,6-anhydro-α-d-galactose, joined by α-(1,3) and β-(1,4) glycosidic linkages [48]. There are three most important types of commercially available carrageenan: monosulfated κ-carrageenan, bisulfated ι-carrageenan and trisulfated λ-carrageenan, however only the first two can form gels [49]. The gelation of κ-carrageenan occurs in the presence of monovalent or divalent cations and their mixtures upon cooling [50]. The κ-carrageenan hydrogels are thermo-sensitive and undergo reversible volume transitions in response to thermal stimuli, therefore they are suitable materials for delivery systems in which release could be controlled with temperature [51].

The κ-carrageenan hydrogels have been used for encapsulation of bioactive components, such as enzymes [52], antioxidants [53], or probiotics, which might be sensitive to environmental conditions occurring during manufacture and shelf life of the food products or during passage through the gastrointestinal tract. Strains of lactic acid bacteria have been encapsulated into κ-carrageenan gel beads, prepared using KCl as the cross-linker, and lyophilized. The freeze-drying process as well as the rehydration process can be harmful to cells. In mentioned studies, κ-carrageenan matrix allowed maintaining viability of probiotic bacteria during lyophilization and protected cells from osmotic shock during rehydration. In addition, survival rate of encapsulated probiotic has been examined during one-month-long storage at 4 °C and 22 °C (refrigeration conditions and room temperature). Survival rate of all tested strains was high during the treatment and cells maintained their activity [54]. Moreover, probiotic bacteria, such as *L. rhamnosus*, *B. longum*, or *L. acidophilus*, entrapped in κ-carrageenan microcapsules showed higher acid and bile tolerance than the free cells [55].

Properties of hydrogels made of single polysaccharide might be further enhanced by adding a second component, for example κ-carrageenan has been combined with ι-carrageenan and used for encapsulation of *L. acidophilus*. Microcapsules with probiotics were subjected to solutions simulating changing pH conditions during passage through the gastrointestinal tract. Viability of encapsulated bacteria has been maintained after treatment with solutions with various pH. It has been concluded that both polysaccharides used to prepare microcapsules, could create the Interpenetrating Network. The close interaction between entangled chains creating this network has influence on decreasing porosity of hydrogel. Consequently, the diffusion of harmful medium into microcapsules beads matrix has been limited [56].

Bacteria have been entrapped in hydrogel made of polysaccharide not only to improve their viability under conditions of the gastrointestinal tract or storage conditions but also to test their usability in fermenting dairy products. For example, κ-carrageenan–locust bean gum beads with lactic acid bacteria have been successfully used during fermentation of whey-based media [57,58]. The locust bean gum is a galactomannan polysaccharide, consisting of a mannose backbone with galactose side groups, obtained from the locust tree seeds [59].

κ-carrageenan–locust bean gum hydrogels have been used to prepare films containing *L. rhamnosus* probiotics. Storage ability of such films has been tested at 4 °C and 25 °C (storage in a refrigerator and at room temperature) and compared with storage ability of films made of other biomaterials, such as alginate and pectin. It was found that κ-carrageenan–locust bean gum films showed the highest ability to stabilize live probiotic organisms under tested conditions [60].

In the development of delivery systems of probiotics, κ-carrageenan has been combined with other biomaterials, such as DNA. Hydrogels prepared from single-stranded DNA extracted from salmon milt, combined with gelatin and κ-carrageenan, have been used to improve survival of selected probiotic strains (*Lactobacillus*, *Lactococcus* and *Bifidobacterium*) under simulated gastric juice conditions and during long refrigerated storage. In addition, the food-grade hydrogels have been prepared (using commercially food-graded biomaterials) and tested under the simulated gastric conditions. Thus, the potential usability of such hydrogels as delivery systems of probiotics, which could be orally administered, has been confirmed [61].

Interestingly, there are controversies regarding to negative influence of carrageenan on gastrointestinal tract, e.g., it is considered a cause of colitis [62]. It was found that the intake of carrageenan could change the composition of the intestine microbiota: in presence of this polysaccharide, the amount of some species decreased, while at the same time the abundance of other species increased. Carrageenan significantly decreased the populations of *A. muciniphila* and loss of this anti-inflammatory intestine bacteria is significantly relevant for the development of carrageenan-induced colitis. It should be taken into consideration during development of probiotic delivery systems based on carrageenan [63].

The aforementioned κ-carrageenan-based hydrogels used as probiotics delivery systems are placed in Table 2, which summarizes tested probiotic strains as well as type of tested conditions. The κ-carrageenan-based hydrogels improve survival rate of encapsulated probiotic cells not only under gastrointestinal conditions, but also during storage at various temperatures. Probiotics, to fulfill their role, have to be released from hydrogels. Release of probiotic cells from carrageenan-based hydrogels have occurred under simulated intestinal juice conditions. However, rate of probiotic release from carrageenan hydrogels might be slower than from alginate hydrogels. It might be related to fact that carrageenan hydrogels dissolve at a significantly slower rate in the simulated intestinal juice [64].

### 2.3. Xanthan-Based Hydrogels

Xanthan is a branched polysaccharide, the backbone of which consists of β-(1,4) linked d-glucose units with side chains consisting of d-glucuronic acid unit between two d-mannose units attached to every second glucose residue in the backbone [65]. Xanthan is produced via fermentation by bacteria from, e.g., agro-industrial wastes such as straw, corn cobs or fruit peels [66]. Hydrogels based on xanthan can be formed in the presence of bivalent cations, such as Ca^2+^, Mg^2+^, Cd^2+^ or Pb^2+^ [67,68]. Xanthan and hydrogels based on this polysaccharide have found applications in various fields, such as medicine (e.g., tissue engineering and drug delivery systems) [69,70] or food industry (e.g., freeze–thaw stabilizers) [71,72].

Xanthan and alginate have been used as materials for microencapsulation of *L. plantarum*. To establish the pH tolerance, alginate–xanthan beads with probiotic cells, as well as free cells, have been subjected to simulated gastric and intestinal fluids. The survivability of encapsulated probiotic cells after contact with low gastric pH was higher than survivability of free cells. Moreover, it was found that coating of alginate–xanthan beads with chitosan further enhanced survivability of encapsulated cells under acidic conditions. Under simulated intestinal conditions, the uncoated alginate–xanthan beads disintegrated in less than an hour, while coated beads required twice as much time to achieve maximum cell release. However, both types of beads were able to provide sufficient viable probiotics to the targeted site. Furthermore, thermotolerance of probiotic cells encapsulated into coated and uncoated beads has been tested during heat treatment of 75 °C for 30 s and 90 °C for 5 s. It was established that polysaccharide beads, acting as heat barrier, improved heat tolerance of *L. plantarum* bacteria under tested conditions [73].

Xanthan, the anionic polysaccharide, can form physical hydrogels with cationic chitosan. A strain of probiotic bacteria *Pediococcus acidilactici* was encapsulated into such hydrogels and exposed to the simulated gastrointestinal conditions. The encapsulated *P. acidilactici* under simulated gastric fluids showed higher survival rate than free cells subjected to those conditions. Moreover, release from xanthan–chitosan hydrogels was examined, and it was found that the probiotics release was negligible under gastric low pH, while a complete release occurred under intestinal conditions. This desired behavior is related to pH-sensitive swelling of xanthan–chitosan hydrogels. Additionally, in presented studies, the positive effect of encapsulation on the viability of probiotic cells after freeze-drying was observed [74]. Xanthan–chitosan hydrogels have been used to encapsulate other probiotic strains, for example *L. acidophilus*. The solutions with different concentrations of both polysaccharides, as well as different cells to polysaccharides ratios, have been tested to establish optimal encapsulation conditions [75].

Xanthan–chitosan hydrogels could be used to improve survival of encapsulated probiotics not only under gastrointestinal conditions but also during storage in yogurt under conditions corresponding to storage in a refrigerator and at room temperature. Two kinds of beads, single-layer (xanthan–chitosan) and double-layer (xanthan–chitosan–xanthan), were prepared. Both types of beads with probiotic cells were kept in yogurt for 21 days at 4 °C and 25 °C. The survival rate of *B. bifidum* cells encapsulated in both types of beads in storage at 4 °C has been improved significantly in comparison to free probiotic cells. Although the survival of encapsulated cells at 25 °C was higher than survival of free cells at that temperature, the level of viable cells was below the level recommended by World Health Organization. In addition, the release of probiotic cells from both types of beads has been examined in the presence of simulated intestinal fluids and the single-layer beads showed better release profile under tested conditions [76]. The xanthan–chitosan and xanthan–chitosan–xanthan beads have been tested as encapsulation systems for other probiotic strains, e.g., *L. acidophilus*. During this study it was confirmed once again that encapsulation in beads based on xanthan enhanced survival of the probiotic cells in yogurt during storage at 4 °C and 25 °C for 21 days [77].

The aforementioned xanthan-based hydrogels used as probiotic delivery systems are placed in Table 3, along with encapsulated probiotic strains and type of tested conditions. Xanthan-based hydrogels were proven to be capable of improving probiotic cells viability, e.g., during storage in yogurt.

### 2.4. Pectin-Based Hydrogels

Pectin, an anionic polysaccharide, consists of a linear backbone of α-(1-4) linked d-galacturonic acid which can be partially methylated [78]. As it is a component of plants cell wall, pectin can be extracted from citrus peels, apple pomace, sugar beet, pumpkin pulp or potato pulp [79,80,81]. Pectin hydrogels can be obtained in the presence of crosslinking bivalent cations, such as Ca^2+^ [82]. Pectin, as well as hydrogels made of this polysaccharide, might be used in medicine related applications, e.g., as drug delivery systems [83] or in food industry, e.g., as texture modifiers or fat replacers [84].

Selected probiotic strain, *Lactobacillus rhamnosus*, has been encapsulated into pectin hydrogel beads. Additionally, glucose has been introduced into part of the beads. Beads with probiotics were subjected to simulated gastric fluid and simulated colonic fluid containing enzymes. Encapsulation provides good protection for probiotic cells against acidic conditions and enzymes. Moreover, the addition of glucose further improved their protective abilities. Before the beads with encapsulated probiotic cells were subjected to gastrointestinal conditions, they were freeze-dried and stored at room temperature for over one month. The survival rate of encapsulated probiotic cells during freeze-drying has been improved in comparison to free cells. It is worth noting that introduction of glucose into pectin beads further improved cells survivability during lyophilization. As could be predicted, shelf life of encapsulated cells was longer than shelf life of free cells stored at ambient temperature [85]. Glucose has a positive influence on viability of probiotic cells; e.g., as cryoprotectant agent, it can inhibit formation of ice crystals which are harmful to cells [86]. Furthermore, the viability of *L. rhamnosus* subjected to simulated gastric fluids has been improved in the presence of glucose [87].

Properties of pectin hydrogels, for applications as probiotic delivery systems, could be further improved by coating them with chitosan. *L. casei* cells were encapsulated into pectin hydrogel beads, and then the beads were coated with chitosan. Viability of cells encapsulated into coated and uncoated beads, as well as viability of free cells, was investigated under simulated gastric fluid. Encapsulation in pectin and pectin–chitosan beads improved cells survivability under acidic conditions. Moreover, this research allowed establishing the effect of coating of pectin beads with chitosan. It was found that coating with chitosan has a significantly positive influence on viability of bacteria under simulated gastric conditions. Chitosan-coated beads with probiotic bacteria were also subjected to simulated intestinal conditions. As a result of disintegration of coated pectin beads under simulated intestinal fluid, the release of viable probiotic cells from beads occurred after one hour [88]. As is known, providing high numbers of living probiotic cells into the intestines contributes to their proper functioning.

Pectin, similar to other polysaccharides mentioned above, could be used in combination with non-polysaccharide polymers to form probiotic delivery systems. Pectin hydrogel microparticles were obtained in the presence of calcium hydrochloride and a part of microparticles was coated with whey protein. The interaction between protonated amine groups of whey protein and negatively charged carboxylic groups of pectin occurred during coating. Coated and uncoated microparticles with encapsulated probiotic strain of *L. acidophilus*, have been exposed to simulated gastric and intestinal fluids. Coated and uncoated microparticles subjected to gastric acid conditions remained intact and the viability of encapsulated *L. acidophilus* was maintained at higher level than viability of free probiotic cells. Interestingly, the coating of the particles with whey proteins did not provide additional protection to encapsulated probiotic cells. Although uncoated microparticles exposed to intestinal conditions remained intact, the coated microparticles disintegrated after being subjected to intestinal fluids allowing the release of probiotic cells [89]. In another study, a different probiotic strain, *Lactobacillus rhamnosus*, was encapsulated into pectin hydrogel beads coated with whey protein. Such beads provided good protection of encapsulated cells against gastric fluid. However, in that study, the survival of the probiotic bacteria encapsulated into uncoated pectin beads was not tested, so effect of coating with whey protein on cells survivability could not be clearly defined [90].

In other study, pectin has been used with rice bran extract to encapsulate probiotic cells. The rice bran extract contains carbohydrates, protein and fat. The *L. plantarum* encapsulated in pectin and pectin–rice bran extract beads, as well as free probiotic cells, have been subjected to acidic and bile conditions. Pectin beads improved the survival rate of encapsulated cells in comparison to free cells subjected to the same conditions. Moreover, it was established that the rice bran extract helped to further enhance the viability of probiotic cells after exposure to harmful conditions [91].

Pectin-based hydrogels used as delivery systems for selected probiotic strains along with information concerning type of tested conditions were summarized in Table 4.

### 2.5. Chitosan-Based Hydrogels

Chitosan is an aminopolysaccharide derived from chitins, composed of β-(1,4) linked d-glucosamine and *N*-acetyl-d-glucosamine [36]. Chitosan, as it was described above, has been successfully used in combination with other polysaccharides as probiotic delivery systems. Layer of chitosan has positive influence on probiotic delivery systems protection abilities against harmful conditions. Chitosan-based hydrogels have been used for numerous applications, such as drug delivery systems [92], bone regeneration materials [93] or wound dressings [94]. However, chitosan, as the only cationic polysaccharide of natural origin, shows antimicrobial properties [95]. Those antimicrobial properties are related to strong electrostatic interaction between positively charged chitosan and negatively charged cell surface of bacteria. Consequently, it causes changes in the functioning of cell membrane accompanied by increased membrane permeability, which leads to destabilization of cell membrane and leakage of intracellular substances, and finally to the death of the cell [96]. Therefore, chitosan cannot be used as a solitary material for creating hydrogels for probiotic delivery systems.

## 3. Conclusions

Possibilities of efficient applications of polysaccharide hydrogels as probiotic delivery systems have been proven in numerous studies. Those hydrogels can improve the survival of encapsulated probiotic strains under gastrointestinal track conditions, as well as during storage at various temperatures or during heat treatment. Table 1, Table 2, Table 3 and Table 4 provide a summary of the types of tested hydrogels, used probiotic strains, and research conditions. However, the best delivery system among all listed in this review cannot be indicated. Each of the cited studies was carried out under individual conditions (temperature, pH, and time), using different probiotic strains having various resistances to enzymes or acidic conditions. Therefore, based on comparison of the results of those studies, it is not possible to clearly indicate the most efficient hydrogel.

Many polysaccharide-based hydrogels have tested as probiotic delivery systems and the list is constantly increasing. Therefore, only selected hydrogels were described above to indicate the possibilities of using them to design probiotic delivery systems suitable for certain bacteria strains and for various conditions. Based on cited studies, it can be noticed that hydrogels obtained from mixture of two polysaccharides or polysaccharide and non-polysaccharide compounds provide better protection against tested conditions than hydrogels made of single polysaccharides. Addition of a second component into probiotic delivery systems, especially as coating, might decrease the size of pores and, as a result, limit the contact between probiotic cells and a harmful medium. Based on research results cited in this review, bi-component polysaccharide-based hydrogels are more suitable as delivery systems for probiotics and it should be considered during development of new probiotic delivery systems.

## Figures and Tables

**Table 1:** Alginate-based hydrogels used as delivery systems of selected probiotic strains.

Biopolymer(s)	Probiotic Strains	Tested Conditions	Ref.
Alginate	*B. breve*	gastric fluids	[32]
Alginate, alginate–chitosan	*L. rhamnosus*, *L. acidophilus*	gastric fluids, bile salts	[37]
Alginate–chitosan	*L. plantarum*	gastric fluids, bile salts, pancreatic enzymes	[38]
Alginate, alginate–chitosan	*L. plantarum*	storage in pomegranate juice at fridge	[40]
Alginate	*L. rhamnosus*, *B. longum*, *L. salivarius*, *L. plantarum*, *L. acidophilus*, *L. paracasei*, *B. lactis*	gastric fluids and bile salts; heat treatment	[42]
Alginate, alginate–whey proteins	*L. plantarum*	gastric fluids, bile salts, pancreatic enzymes	[44]
Alginate–pea protein isolate	*L. casei*	storage at room temp., fridge and freezer	[45]
Alginate, alginate–gelatin	*L. salivarious*	wet storage; heat treatment; saliva, gastric fluids and bile salts	[47]

**Table 2:** κ-Carrageenan-based hydrogels used as delivery systems of selected probiotic strains.

Biopolymer(s)	Probiotic Strains	Tested Conditions	Ref.
κ-carrageenan	*L. delbrueckii*, *L. casei*, *L. lactis*, *S. thermophilus*	freeze-drying; storage at room temp. and fridge	[54]
κ-carrageenan	*L. rhamnosus*, *B. longum*, *L. salivarius*, *L. plantarum*, *L. acidophilus*, *L. paracasei*, *B. lactis*, *L. rhamnosus*, *B. bifidum*	gastric fluids and bile salts	[55]
κ-carrageenan–ι-carrageenan	*L. acidophilus*	pH conditions of the gastrointestinal tract	[56]
κ-carrageenan–locust bean gum	*L. rhamnosus*	storage at room temp. and fridge	[60]
DNA–gelatin–κ-carrageenan	*B. lactis*, *B. longum*, *B. bifidum*, *L. acidophilus*	gastric fluids; storage at fridge	[61]

**Table 3:** Xanthan-based hydrogels used as delivery systems of selected probiotic strains.

Biopolymer(s)	Probiotic Strains	Tested Conditions	Ref.
Xanthan–alginate, xanthan–alginate–chitosan	*L. plantarum*	gastric fluids and bile salts; heat treatment	[73]
Xanthan–chitosan	*P. acidilactici*	gastric and intestinal fluids; freeze-drying	[74]
Xanthan–chitosan, xanthan–chitosan–xanthan	*B. bifidum*	storage at room temp. and fridge in yogurt; gastric fluids and bile salts	[76]
Xanthan–chitosan, xanthan–chitosan–xanthan	*L. acidophilus*	storage in yogurt at room temp. and fridge	[77]

**Table 4:** Pectin-based hydrogels used as delivery systems of selected probiotic strains.

Biopolymer(s)	Probiotic Strains	Tested Conditions	Ref.
Pectin	*L. rhamnosus*	freeze-drying; stored at room temperature; gastric fluids, enzymes	[85]
Pectin, pectin–chitosan	*L. casei*	gastric and intestinal fluids	[88]
Pectin–whey protein	*L. acidophilus*	gastric and intestinal fluids	[89]
Pectin–whey protein	*L. rhamnosus*	gastric fluid	[90]
Pectin, pectin–rice bran extract	*L. plantarum*	acid and bile solutions	[91]

## References

[1] Augustin M.A., Hemar Y. (2009). Nano- and micro-structured assemblies for encapsulation of food ingredients. Chem. Soc. Rev..

[2] Tamjidi F., Shahedi M., Varshosaz J., Nasirpour A. (2013). Nanostructured lipid carriers (NLC): A potential delivery system for bioactive food molecules. Innov. Food Sci. Emerg. Technol..

[3] Aditya N.P., Espinosa Y.G., Norton I.T. (2017). Encapsulation systems for the delivery of hydrophilic nutraceuticals: Food application. Biotechnol. Adv..

[4] Gbassi G.K., Vandamme T. (2012). Probiotic Encapsulation Technology: From Microencapsulation to Release into the Gut. Pharmaceutics.

[5] Nayak A.K., Das B., Maji R. (2012). Calcium alginate/gum Arabic beads containing glibenclamide: Development and in vitro characterization. Int. J. Biol. Macromol..

[6] FAO/WHO (2006). Probiotics in Food. Health and Nutritional Properties and Guidelines for Evaluation.

[7] Tuohy K.M., Probert H.M., Smejkal C.W., Gibson G.R. (2003). Using probiotics and prebiotics to improve gut health. Drug Discov. Today.

[8] Wang Y. (2009). Prebiotics: Present and future in food science and technology. Food Res. Int..

[9] Lye H.-S., Kuan C.-Y., Ewe J.-A., Fung W.-Y., Liong M.-T. (2009). The Improvement of Hypertension by Probiotics: Effects on Cholesterol, Diabetes, Renin, and Phytoestrogens. Int. J. Mol. Sci..

[10] Rautava S., Kalliomäki M., Isolauri E. (2002). Probiotics during pregnancy and breastfeeding might confer immunomodulatory protection against atopic disease in the infant. J. Allergy Clin. Immunol..

[11] Liong M.T. (2008). Roles of Probiotics and Prebiotics in Colon Cancer Prevention: Postulated Mechanisms and In-vivo Evidence. Int. J. Mol. Sci..

[12] Aragón F., Perdigón G., de Moreno de LeBlanc A. (2014). Modification in the diet can induce beneficial effects against breast cancer. World J. Clin. Oncol..

[13] Govender M., Choonara Y.E., Kumar P., du Toit L.C., van Vuuren S., Pillay V. (2014). A Review of the Advancements in Probiotic Delivery: Conventional vs. Non-conventional Formulations for Intestinal Flora Supplementation. AAPS PharmSciTech.

[14] Varankovich N.V., Nickerson M.T., Korber D.R. (2015). Probiotic-based strategies for therapeutic and prophylactic use against multiple gastrointestinal diseases. Front. Microbiol..

[15] McClements D.J. (2017). Designing biopolymer microgels to encapsulate, protect and deliver bioactive components: Physicochemical aspects. Adv. Colloid Interface Sci..

[16] Tripathi M.K., Giri S.K. (2014). Probiotic functional foods: Survival of probiotics during processing and storage. J. Funct. Foods.

[17] Corona-Hernandez R.I., Alvarez-Parrilla E., Lizardi-Mendoza J., Islas-Rubio A.R., de la Rosa L.A., Wall-Medrano A. (2013). Structural stability and viability of microencapsulated probiotic bacteria: A review. Compr. Rev. Food Sci. Food Saf..

[18] Cook M.T., Tzortzis G., Charalampopoulos D., Khutoryanskiy V.V. (2012). Microencapsulation of probiotics for gastrointestinal delivery. J. Control. Release.

[19] Kim J., Muhammad N., Jhun B.H., Yoo J.W. (2016). Probiotic delivery systems: A brief overview. J. Pharm. Investig..

[20] De Barros J.M., Scherer T., Charalampopoulos D., Khutoryanskiy V.V., Edwards A.D. (2014). A laminated polymer film formulation for enteric delivery of live vaccine and probiotic bacteria. J. Pharm. Sci..

[21] Solanki H.K., Pawar D.D., Shah D.A., Prajapati V.D., Jani G.K., Mulla A.M., Thakar P.M. (2013). Development of Microencapsulation Delivery System for Long-Term Preservation of Probiotics as Biotherapeutics Agent. Biomed. Res. Int..

[22] Cook M.T., Charalampopoulos D., Khutoryanskiy V.V., Connon C.J., Hamley I.W. (2014). Microencapsulation of Probiotic Bacteria into Alginate Hydrogels. Hydrogels in Cell-Based Therapies.

[23] O’Sullivan L., Murphy B., McLoughlin P., Duggan P., Lawlor P.G., Hughes H., Gardiner G.E. (2010). Prebiotics from Marine Macroalgae for Human and Animal Health Applications. Mar. Drugs.

[24] Okolie C.L., Rajendran S.R.C.K., Udenigwe C.C., Aryee A.N.A., Mason B. (2017). Prospects of brown seaweed polysaccharides (BSP) as prebiotics and potential immunomodulators. J. Food Biochem..

[25] Urtuvia V., Maturana N., Acevedo F., Peña C., Díaz-Barrera A. (2017). Bacterial alginate production: An overview of its biosynthesis and potential industrial production. World. J. Microbiol. Biotechnol..

[26] Sun J., Tan H. (2013). Alginate-Based Biomaterials for Regenerative Medicine Applications. Materials.

[27] Lee K.Y., Mooney D.J. (2012). Alginate: Properties and biomedical applications. Prog. Polym. Sci..

[28] Draget K.I., Taylor C. (2011). Chemical, physical and biological properties of alginates and their biomedical implications. Food Hydrocoll..

[29] Harnsilawat T., Pongsawatmanit R., McClements D.J. (2006). Characterization of β-lactoglobulin–sodium alginate interactions in aqueous solutions: A calorimetry, light scattering, electrophoretic mobility and solubility study. Food Hydrocoll..

[30] Matyash M., Despang F., Ikonomidou C., Gelinsky M. (2014). Swelling and Mechanical Properties of Alginate Hydrogels with Respect to Promotion of Neural Growth. Tissue Eng. Part C.

[31] Bajpai S.K., Kirar N. (2016). Swelling and drug release behavior of calcium alginate/poly (sodium acrylate) hydrogel beads. Des. Monomers Polym..

[32] Li Y., Feng C., Li J., Mu Y., Liu Y., Kong M., Cheng X., Chen X. (2017). Construction of multilayer alginate hydrogel beads for oral delivery of probiotics cells. Int. J. Biol. Macromol..

[33] Mokarram R.R., Mortazavi S.A., Najafi M.B.H., Shahidi F. (2009). The influence of multi stage alginate coating on survivability of potential probiotic bacteria in simulated gastric and intestinal juice. Food Res. Int..

[34] Li Y., Kong M., Feng C., Liu W.F., Liu Y., Cheng X.J., Chen X.G. (2012). Preparation and property of layer-by-layer alginate hydrogel beads based on multi-phase emulsion technique. J. Sol-Gel Sci. Technol..

[35] Muthukumarasamy P., Allan-Wojtas P., Holley R.A. (2006). Stability of Lactobacillus reuteri in Different Types of Microcapsules. J. Food Sci..

[36] Periayah M.H., Halim A.S., Saad A.Z.M. (2016). Chitosan: A Promising Marine Polysaccharide for Biomedical Research. Pharmacogn. Rev..

[37] Sohail A., Turner M.S., Coombes A., Bostrom T., Bhandari B. (2011). Survivability of probiotics encapsulated in alginate gel microbeads using a novel impinging aerosols method. Int. J. Food Microbiol..

[38] Zaeim D., Sarabi-Jamab M., Ghorani B., Kadkhodaee R., Tromp R.H. (2017). Electrospray assisted fabrication of hydrogel microcapsules by single- and double-stage procedures for encapsulation of probiotics. Food Bioprod. Process..

[39] Perricone M., Bevilacqua A., Altieri C., Sinigaglia M., Corbo M.R. (2015). Challenges for the Production of Probiotic Fruit Juices. Beverages.

[40] Nualkaekul S., Lenton D., Cook M.T., Khutoryanskiy V.V., Charalampopoulos D. (2012). Chitosan coated alginate beads for the survival of microencapsulated Lactobacillus plantarum in pomegranate juice. Carbohydr. Polym..

[41] Petruzzi L., Campaniello D., Speranza B., Corbo M.R., Sinigaglia M., Bevilacqua A. (2017). Thermal Treatments for Fruit and Vegetable Juices and Beverages: A Literature Overview. Compr. Rev. Food Sci. Food Saf..

[42] Ding W.K., Shah N.P. (2007). Shah. Acid, Bile, and Heat Tolerance of Free and Microencapsulated Probiotic Bacteria. J. Food Sci..

[43] Khem S., Small D.M., May B.K. (2016). The behaviour of whey protein isolate in protecting Lactobacillus plantarum. Food Chem..

[44] Gbassi G.K., Vandamme T., Ennahar S., Marchioni E. (2009). Microencapsulation of Lactobacillus plantarum spp in an alginate matrix coated with whey proteins. Int. J. Food Microbiol..

[45] Xu M., Gagné-Bourque F., Dumont M.-J., Jabaji S. (2016). Encapsulation of Lactobacillus casei ATCC 393 cells and evaluation of their survival after freeze-drying, storage and under gastrointestinal conditions. J. Food Eng..

[46] Hofman K., Tucker N., Stanger J., Staiger M., Marshall S., Hall B. (2012). Effects of the molecular format of collagen on characteristics of electrospun fibres. J. Mater. Sci..

[47] Yao M., Wu J., Li B., Xiao H., McClements D.J., Li L. (2017). Microencapsulation of Lactobacillus salivarious Li01 for enhanced storage viability and targeted delivery to gut microbiota. Food Hydrocoll..

[48] Dos Santos M.A., Grenha A., Donev R. (2015). Polysaccharide Nanoparticles for Protein and Peptide Delivery: Exploring Less-Known Materials. Advances in Protein Chemistry and Structural Biology.

[49] Kariduraganavar M.Y., Kittur A.A., Kamble R.R., Kumbar S., Laurencin C., Deng M. (2014). Polymer Synthesis and Processing. Natural and Synthetic Biomedical Polymers.

[50] Nguyen B.T., Nicolai T., Benyahia L., Chassenieux C. (2014). Synergistic effects of mixed salt on the gelation of κ-carrageenan. Carbohydr. Polym..

[51] Daniel-da-Silva A.L., Ferreira L., Gil A.M., Trindade T. (2011). Synthesis and swelling behavior of temperature responsive κ-carrageenan nanogels. J. Colloid Interface Sci..

[52] Zhang Z., Zhang R., Chen L., McClements D.J. (2016). Encapsulation of lactase (β-galactosidase) into κ-carrageenan-based hydrogel beads: Impact of environmental conditions on enzyme activity. Food Chem..

[53] Zhang Z., Zhang R., Zou L., Chen L., Ahmed Y., Al Bishri W., Balamash K., McClements D.J. (2016). Encapsulation of curcumin in polysaccharide-based hydrogel beads: Impact of bead type on lipid digestion and curcumin bioaccessibility. Food Hydrocoll..

[54] Denkova Z., Krastanov A., Murgov I. (2004). Immobilized lactic acid bacteria for application as dairy starters and probiotic preparations. J. Gen. Appl. Microbiol..

[55] Ding W.K., Shah N.P. (2009). Effect of Various Encapsulating Materials on the Stability of Probiotic Bacteria. J. Food Sci..

[56] Setijawati D. (2014). Carrageenan from Eucheuma sp and concentration difference as encapsulation material toward Lactobacillus acidophilus viability at simulation GI Tract pH condition. J. Basic Appl. Sci. Res..

[57] Lamboley L., Lacroix C., Champagne C.P., Vuillemard J.C. (1997). Continuous mixed strain mesophilic lactic starter production in supplemented whey permeate medium using immobilized cell technology. Biotechnol. Bioeng..

[58] Doleyres Y., Fliss I., Lacroix C. (2004). Continuous Production of Mixed Lactic Starters Containing Probiotics Using Immobilized Cell Technology. Biotechnol. Prog..

[59] Meunier L., Garthoff J.A., Schaafsma A., Krul L., Schrijver J., van Goudoever J.B., Speijers G., Vandenplas Y. (2014). Locust bean gum safety in neonates and young infants: An integrated review of the toxicological database and clinical evidence. Regul. Toxicol. Pharmacol..

[60] Soukoulis C., Behboudi-Jobbehdar S., Macnaughtan W., Parmenter C., Fisk I.D. (2017). Stability of Lactobacillus rhamnosus GG incorporated in edible films: Impact of anionic biopolymers and whey protein concentrate. Food Hydrocoll..

[61] Jonganurakkun B., Nodasaka Y., Sakairi N., Nishi N. (2006). DNA-Based Gels for Oral Delivery of Probiotic Bacteria. Macromol. Biosci..

[62] Ariffin S.H., Yeen W.W., Abidin I.Z., Abdul Wahab R.M., Ariffin Z.Z., Senafi S. (2014). Cytotoxicity effect of degraded and undegraded kappa and iota carrageenan in human intestine and liver cell lines. BMC Complement. Altern. Med..

[63] Shang Q., Sun W., Shan X., Jiang H., Cai C., Hao J., Li G., Yu G. (2017). Carrageenan-induced colitis is associated with decreased population of anti-inflammatory bacterium, Akkermansia muciniphila, in the gut microbiota of C57BL/6J mice. Toxicol. Lett..

[64] Cheow W.S., Hadinoto K. (2013). Biofilm-Like Lactobacillus rhamnosus Probiotics Encapsulated in Alginate and Carrageenan Microcapsules Exhibiting Enhanced Thermotolerance and Freeze-Drying Resistance. Biomacromolecules.

[65] Tavares F.C., Dorr D.S., Pawlicka A., Avellaneda C.O. (2018). Microbial origin xanthan gum-based solid polymer electrolytes. J. Appl. Polym. Sci..

[66] Dos Santos F.P., Oliveira A.M., Nunes T.P., de Farias Silva C.E., de Souza Abud A.K. (2016). Bioconversion of Agro-industrial Wastes into Xanthan Gum. Chem. Eng. Trans..

[67] Dário A.F., Hortêncio L.M.A., Sierakowski M.R., Neto J.C.Q., Petri D.F.S. (2011). The effect of calcium salts on the viscosity and adsorption behavior of xanthan. Carbohydr. Polym..

[68] Bueno V.B., Bentini R., Catalani L.H., Petri D.F.S. (2013). Synthesis and swelling behavior of xanthan-based hydrogels. Carbohydr. Polym..

[69] Tao Y.Z., Tang S.M., Chen Z., Qiu R.G., Li Q., Du S.M., You Z.L. (2017). Injectable and release-controlled hydrogel based on xanthan gum and silk fibroin. J. Control. Release.

[70] Huang J.J., Deng Y.M., Ren J.A., Chen G.P., Wang G.F., Wang F., Wu X.W. (2018). Novel in situ forming hydrogel based on xanthan and chitosan re-gelifying in liquids for local drug delivery. Carbohydr. Polym..

[71] Muadklay J., Charoenrein S. (2008). Effects of hydrocolloids and freezing rates on freeze–thaw stability of tapioca starch gels. Food Hydrocoll..

[72] Shiroodi S.G., Rasco B.A., Lo Y.M. (2015). Influence of Xanthan-Curdlan Hydrogel Complex on Freeze-Thaw Stability and Rheological Properties of Whey Protein Isolate Gel over Multiple Freeze-Thaw Cycle. J. Food Sci..

[73] Fareez I.M., Lim S.M., Mishra R.K., Ramasamy K. (2015). Chitosan coated alginate–xanthan gum bead enhanced pH and thermotolerance of Lactobacillus plantarum LAB12. Int. J. Biol. Macromol..

[74] Argin S., Kofinas P., Lo Y.M. (2014). The cell release kinetics and the swelling behavior of physically crosslinked xanthan-chitosan hydrogels in simulated gastrointestinal conditions. Food Hydrocoll..

[75] Chen H., Song Y., Liu N., Wan H., Shu G., Liao N. (2015). Effect of complexation conditions on microcapsulation of Lactobacillus acidophilus in xanthan-chitosan polyelectrolyte complex gels. Acta Sci. Pol. Technol. Aliment..

[76] Chen L., Yang T., Song Y., Shu G., Chen H. (2017). Effect of xanthan-chitosan-xanthan double layer encapsulation on survival of Bifidobacterium BB01 in simulated gastrointestinal conditions, bile salt solution and yogurt. LWT Food Sci. Technol..

[77] Shu G., He Y., Chen L., Song Y., Meng J., Chen H. (2017). Microencapsulation of Lactobacillus Acidophilus by Xanthan-Chitosan and Its Stability in Yoghurt. Polymers.

[78] Moreira M.M., Guido L.F., Cruz J.M., Barros A.A. (2010). Determination of galacturonic acid content in pectin from fruit juices by liquid chromatographydiode array detection-electrospray ionization tandem mass spectrometry. Cent. Eur. J. Chem..

[79] Ciriminna R., Fidalgo A., Delisi R., Ilharco L.M., Pagliaro M. (2016). Pectin Production and Global Market. Agro Food Ind. Hi-Tech..

[80] Ptichkina N.M., Markina O.A., Rumyantseva G.N. (2008). Pectin extraction from pumpkin with the aid of microbial enzymes. Food Hydrocoll..

[81] Yang J.S., Mu T.H., Ma M.M. (2018). Extraction, structure, and emulsifying properties of pectin from potato pulp. Food Chem..

[82] Moreira H.R., Munarin F., Gentilini R., Visai L., Granja P.L., Tanzi M.C., Petrini P. (2014). Injectable pectin hydrogels produced by internal gelation: pH dependence of gelling and rheological properties. Carbohydr. Polym..

[83] Jung J., Arnold R.D., Wicker L. (2013). Pectin and charge modified pectin hydrogel beads as a colon-targeted drug delivery carrier. Colloids Surf. B.

[84] Lim J., Ko S., Lee S. (2014). Use of *Yuja* (*Citrus junos*) Pectin as a Fat Replacer in Baked Foods. Food Sci. Biotechnol..

[85] Li R., Zhang Y., Polk D.B., Tomasula P.M., Yan F., Liu L.S. (2016). Preserving viability of Lactobacillus rhamnosus GG in vitro and in vivo by a new encapsulation system. J. Control. Release.

[86] Pollock K., Yu G.L., Moller-Trane R., Koran M., Dosa P.I., McKenna D.H., Hubel A. (2016). Combinations of Osmolytes, Including Monosaccharides, Disaccharides, and Sugar Alcohols Act in Concert During Cryopreservation to Improve Mesenchymal Stromal Cell Survival. Tissue Eng. Part C.

[87] Corcoran B.M., Stanton C., Fitzgerald G.F., Ross R.P. (2005). Survival of Probiotic Lactobacilli in Acidic Environments Is Enhanced in the Presence of Metabolizable Sugars. Appl. Environ. Microbiol..

[88] Bepeyeva A., de Barros J.M.S., Albadran H., Kakimov A.K., Kakimova Z.K., Charalampopoulos D., Khutoryanskiy V.V. (2017). Encapsulation of Lactobacillus casei into Calcium Pectinate-Chitosan Beads for Enteric Delivery. J. Food Sci..

[89] Gebara C., Chaves K.S., Ribeiro M.C.E., Souza F.N., Grosso C.R.F., Gigante M.L. (2013). Viability of Lactobacillus acidophilus La5 in pectin–whey protein microparticles during exposure to simulated gastrointestinal conditions. Food Res. Int..

[90] Gerez C.L., de Valdez G.F., Gigante M.L., Grosso C.R.F. (2012). Whey protein coating bead improves the survival of the probiotic Lactobacillus rhamnosus CRL 1505 to low pH. Lett. Appl. Microbiol..

[91] Chotiko A., Sathivel S. (2016). Development of a combined low-methoxyl-pectin and rice-bran extract delivery system to improve the viability of Lactobacillus plantarum under acid and bile conditions. LWT Food Sci. Technol..

[92] Bhattarai N., Gunn J., Zhang M. (2010). Chitosan-based hydrogels for controlled, localized drug delivery. Adv. Drug Deliv. Rev..

[93] Lišková J., Douglas T.E., Beranová J., Skwarczyńska A., Božič M., Samal S.K., Modrzejewska Z., Gorgieva S., Kokol V., Bačáková L. (2015). Chitosan hydrogels enriched with polyphenols: Antibacterial activity, cell adhesion and growth and mineralization. Carbohydr. Polym..

[94] Liu H., Wang C., Li C., Qin Y., Wang Z., Yang F., Li Z., Wang J. (2018). A functional chitosan-based hydrogel as a wound dressing and drug delivery system in the treatment of wound healing. RSC Adv..

[95] Sonia T.A., Sharma C.P. (2011). Chitosan and Its Derivatives for Drug Delivery Perspective. Adv. Polym. Sci..

[96] Kong M., Chen X.G., Xing K., Park H.J. (2010). Antimicrobial properties of chitosan and mode of action: A state of the art review. Int. J. Food. Microbiol..

